# The acrid raphides in tuberous root of *Pinellia ternata* have lipophilic character and are specifically denatured by ginger extract

**DOI:** 10.1007/s11418-020-01425-6

**Published:** 2020-06-26

**Authors:** Tsukasa Fueki, Koichiro Tanaka, Kunihiko Obara, Ryudo Kawahara, Takao Namiki, Toshiaki Makino

**Affiliations:** 1 Matsuya Pharmacy, Niigata, Japan; 2 Department of Traditional Medicine, Toho University School of Medicine, Tokyo, Japan; 3 Department of Pharmacognosy, Graduate School of Pharmaceutical Sciences, Nagoya City University, Nagoya, Japan; 4 Obara Coloproctology Clinic, Tokyo, Japan; 5 Department of Cardiology, Tokyo Nishi Tokushukai Hospital, Tokyo, Japan; 6 Department of Japanese-Oriental (Kampo) Medicine, Graduate School of Medicine, Chiba University, Chiba, Japan

**Keywords:** *Pinellia ternata*, Pinellia tuber, Raphide, Denaturation, Acridity, Ginger

## Abstract

Unprocessed dried tuberous root of *Pinellia ternata* (Pinellia Tuber) has been used as a component of traditional Japanese Kampo medicine, while this crude drug is usually used after processing with Ginger in traditional Chinese medicine (TCM). It is known that the raphides contained in unprocessed Pinellia Tuber can induce severe acrid irritation of the oral and laryngopharynx mucosa when it is boiled insufficiently. However, the mechanism of the inducing irritation by the raphides nor that of the detoxification by heat or processing with Ginger have been left unclear, mainly because of the difficulties in the extraction and the purification of the raphides and in the assays of acridity. In this study, we developed an easy protocol that can be used to collect raphides from unprocessed Pinellia Tuber and an assay protocol that can be used to evaluate the acridity of the raphides in vitro. The raphides of Pinellia Tuber were discovered to have a lipophilic character and to be collected easily by the extraction using petroleum ether. It was also found that the denaturation of the raphides could be assayed by the dispersity of them in petroleum ether layer of the water/petroleum ether partition, and the acridity of the raphides was found to be in correlation with the assayed denaturation. The raphides were denatured by heat, methanol, or Ginger extract and the denaturing activity of Ginger on raphides was found to be attributable to its lipophilic and thermostable components, which may explain the meaning of the processing of Pinellia Tuber with Ginger in TCM, and may lead to the development of an easier and safer protocol to administer Pinellia Tuber. In addition, it was found that rinsing the mouth with salad oil can effectively relieve irritation of the oral mucosa caused by unprocessed Pinellia Tuber, probably due to the lipophilicity of the raphides.

**Electronic supplementary material:**

The online version of this article (10.1007/s11418-020-01425-6) contains supplementary material, which is available to authorized users.

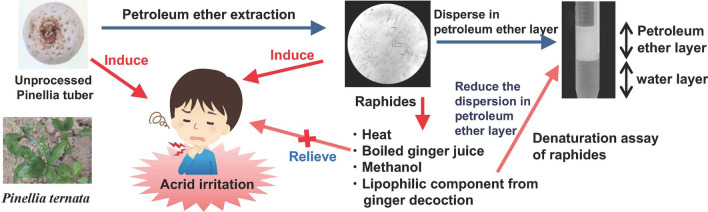

## Introduction

The Japanese Pharmacopoeia defines Pinellia Tuber as the dried tuberous root of *Pinellia ternata*, and describes Pinellia Tuber as having a strong acrid taste [1]. In traditional Japanese Kampo medicine, Pinellia Tuber is used as a component of Kampo formula, such as Shoseiryuto (Xiaoqinglongtang), Hangekobokuto (Banxiahoupotang), and Unkeito (Wenjingtang), and Pinellia Tuber is reported to contain homogentisic acid, 3,4-dihydroxybenzaldehyde, polysaccharides, calcium oxalate, etc. [2]. The Chinese Pharmacopoeia has a similar definition of Pinellia Tuber, and describes it as having a pungent taste with a numbing and irritating sensation, and that its property is warm, pungent, and toxic [3]. Indeed, unprocessed Pinellia Tuber, or its insufficiently boiled decoction, causes acrid irritation of the oral and laryngopharynx mucosa when taken by mistake. Because the irritation is so severe, the Chinese Pharmacopoeia adopts the term “toxic” [3], and the textbook of traditional Chinese medicine categorizes Pinellia Tuber as a toxic substance, like the unprocessed root of *Aconitum carmichaelii* or the seed of *Croton tiglium*, and states that it should be used after detoxification [4]. The Chinese Pharmacopoeia lists three types of processed Pinellia Tuber; prepared Pinellia Tuber refers to dried Pinellia Tuber after decocting in water containing licorice, Pinellia Tuber prepared with alum, which is dried Pinellia Tuber after soaking in alum solution, and Pinellia Tuber prepared with Ginger, which is dried Pinellia Tuber after decocting in water containing Ginger [3, 5]. Conversely, in traditional Japanese Kampo medicine, Pinellia Tuber is not recognized as toxic and is mostly used in an unprocessed form. However, it is recommended that Pinellia Tuber is decocted sufficiently, and preferably with Ginger to remove its acridity.

Many discussions have focused on the acridity of Pinellia Tuber. Homogentisic acid, 3,4-dihydroxybenzaldehyde, and calcium oxalate had been listed as the causing substances of the acridity. However, recent studies strongly suggested that the acridity of Pinellia Tuber was mainly caused by insoluble needle-like crystals, called raphides; these mainly consist of calcium oxalate [6, 7]. Additionally, the raphides were reported to contain protein as well as calcium oxalate, and proteins isolated from the raphides could induce eye inflammation in rabbits without the raphide crystals [8]. It has also been reported on the raphides of taro, a *Colocasia* species, which are similar to those of Pinellia Tuber that the acridity was lost by the protein digestion without any morphological change in the raphides under a light microscope observation, and it was proposed that acridity might be caused by the sharp raphides penetrating the mucous membrane and transporting the inflammatory protein [9].

It is empirically known that the acridity of Pinellia Tuber is decreased by heat, or by processing with alum or Ginger. The interaction of Pinellia Tuber with alum leads to solubilization of the raphides by the exchange of calcium to aluminum in the oxalate crystals [10]. However, very little progress has been made on understanding the mechanism of decreasing acridity by heat and especially via interactions with Ginger. The following factors may have made it difficult to analyze the raphides of Pinellia Tuber: (1) raphide insolubility, (2) difficulty purifying raphides due to the large amount of starch grains in Pinellia Tuber, and (3) the need to assess acridity by performing a gustative bioassay in volunteers, which is not very convenient and forces them to experience irritation.

Referring to a previous report on raphides in taro leaves [9], we established a protocol to purify the raphides of Pinellia Tuber, and obtained data on their characteristics, stability, and their interactions with other crude drugs.

## Materials and methods

### Materials

All crude drugs used were under the quality control of the 17th edition of the Japanese Pharmacopoeia [1]. Pinellia Tuber, the dried root and stolon of *Glycyrrhiza uralensis* (Glycyrrhiza), the dried rhizome of *Cnidium officinale* (Cnidium Rhizome), the dried rhizome of *Atractylodes japonica* (Atractylodes Rhizoma), the dried sclerotium of *Wolfiporia cocos* (Poria), the dried bark of *Magnolia obovata* (Magnoliae Cortex), the dried tips of branches of *Perilla frutescens* var. *crispa* (Perillae Herba), and the dried rhizome of *Zingiber officinale* (dried Ginger) were purchased from Tochimototenkaido (Osaka, Japan), and supplied in cut-form with about 5 mm pieces. The fresh ginger, grown at Kochi prefecture, and salad oil were purchased at a supermarket in Niigata, Japan in 2018.

### Petroleum ether extraction from the raphides in Pinellia Tuber

The sliced dried Pinellia Tuber (25 g) was immersed in purified water (30 ml) for 4 h to absorb water. The liquid water was drained and then petroleum ether (PE, 50 ml) was added and homogenized using an electric mill (Y-308B, Yamamoto Denki, Fukushima, Japan) for 1 min. Cloudy suspension liquid containing raphides was quickly transferred into a beaker leaving the pasty residue in the mill. Fresh PE (50 ml) was added to the residue and re-homogenized using the mill, the liquid was transferred into the same beaker. The procedure was repeated three times, and all liquids were combined. The beaker was kept still for 1 h to let the raphides precipitate. Then the supernatant liquid was removed, and the precipitate was washed twice with fresh PE (40 ml), and then re-suspended in 20 ml of fresh PE and stored at − 20 °C in glass vials. The suspension was named the petroleum ether extraction (PEX) suspension, and was used in the following analysis.

### Preparing decoctions of the crude drugs

Each 6.0 g of Glycyrrhiza, Cnidium Rhizome, Atractylodes Rhizome, or dried Ginger and purified water (32 ml) were sealed in a plastic centrifuge tube (50 ml), and were incubated in boiling water for 30 min, then filtered quickly through a stainless mesh (30 mesh) and stored at 4 °C. The decoctions were used within 24 h after the preparations. A part of the decoction was lyophilized, and the fingerprint patterns of the extracts are shown in Supplemental Figures.

### Extraction of lipophilic components from dried Ginger and Atractylodes Rhizome

PE (1.5 ml) was added to the decoctions (1.5 ml) of dried Ginger or Atractylodes Rhizoma prepared as described above, shaken vigorously, centrifuged at 112 × *g* for 5 min, and then the PE layer was collected. Fresh PE was added to the lower layer, and this procedure was repeated four times; all the PE layers were combined and evaporated by standing in boiling water.

### Preparation of fresh ginger juice

Fresh ginger (105 g) was grated and squeezed. The juice was filtered through cotton gauze, and centrifuged at 1.0 × 10^3^ × *g* for 10 min to precipitate starch grains. The supernatant was divided into two tubes. One tube was sealed and incubated in boiling water for 30 min to prepare boiled ginger juice.

### Reactions of Pinellia Tuber powder and crude drug decoctions, powdered dried Ginger, or ginger juice

Powder of Pinellia Tuber or dried Ginger was prepared by milling them for 1 min using an electric mill (Y-308B, Yamamoto Denki, Fukushima, Japan). Pinellia Tuber powder (0.7 g) and crude drug decoction (2.0 ml) were dispensed into a test tube, and purified water was added to a total volume of 6 ml. After mixing vigorously, the samples were incubated at 4 or 40 °C for 15 min to 2 h. In another experiment, Pinellia Tuber powder (0.7 g) and dried Ginger powder (0.7 g) were mixed and purified water was added to a total volume of 6 ml, then incubated at 40 °C for 2 h. The reaction suspensions were centrifuged at 18 × *g* for 10 min, and the supernatants containing the raphides were collected. Purified water (8 ml) was added to the precipitate, mixed vigorously, re-centrifuged, and the second supernatants were collected and combined with the first. The combined supernatants were centrifuged at 1.0 × 10^3^ × *g* for 10 min to precipitate the residue containing raphides. After removing the supernatant, the residues were re-suspended in purified water (3 ml) for subsequent use in the denaturation assay.

### Raphide denaturation assay

Petroleum ether (3–4 ml) was added to an equal volume of aqueous samples prepared in the above section containing the raphides and shaken vigorously. The samples were kept standing for 15 min at room temperature, and then the upper, middle, and lower layers appeared. Then, the samples were shaken gently to raise the cloud of raphides from the middle layer in the upper layer. Thirty-microliters of the upper layer containing the raphide cloud were collected after 10 s of light shaking and dropped onto a slide glass. The samples were dried and observed using a light microscope at 140× magnification.

### Gustatory test

A gustatory test was performed in three healthy volunteers. The procedures were approved by the ethical committee in Toho University School of Medicine with permission code #A19081. Written informed consent was obtained from all individual participants included in the study. Aqueous sample suspensions (0.5 ml) were kept in the mouth of healthy volunteers for 1 min before being expelled. The acridity was determined after 10 min based on the irritation.

### Preparation of Hangekoubokuto (Banxiahoupotang) immersion

Hangekoubokuto (banxiahoupotang) immersion was prepared using the immersion of powdered crude drugs (IPCD) method [11]. Pinellia Tuber (6.0 g), Poria (5.0 g), Magnolia Bark (3.0 g), Perillae Herba (2.0 g), and dried Ginger (1.0 g) were mixed and milled for 1 min. The powdered crude drugs (7.5 g) were immersed in boiled water (200 ml) and stirred vigorously for 20 s. Then, the immersion was stood for 4 min to precipitate the powder, and the supernatant was poured to prepare the Hangekoubokuto immersion.

## Results

### The extraction of raphides from Pinellia Tuber

**Fig. 1:**
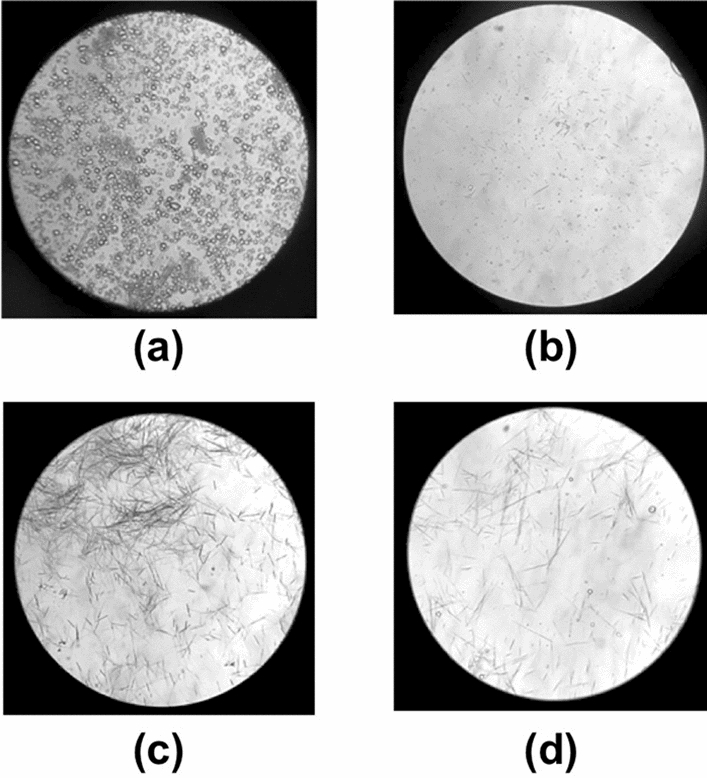
Photos of raphides extracted from Pinellia Tuber. Pinellia Tuber powder (1.0 g) was suspended in purified water (10 ml) and stirred for 10 min (**a**). The suspension was centrifuged at 18 × *g* for 10 min, and the supernatant was collected. Then, the supernatant was centrifuged at 1.0 × 10^3^ × *g* for 10 min and the precipitate was re-suspended in purified water (0.5 ml) (**b**). The original suspension contained many starch grains (**a**), which were partially removed by centrifugation (**b**). The suspension was prepared using the PEX protocol described in “Materials and methods” (**c**). Using the PEX protocol, most of the starch grains were removed, and the raphides were well extracted from Pinellia Tuber (**c**). This suspension was dried and re-suspended in an equal volume of purified water (**d**), which could then be used for the gustatory test. The images were obtained using a light microscope at × 140 magnification

The low-speed centrifugation protocol has been used to isolate raphides from Pinellia Tuber [7]. However, the centrifugation conditions were not clearly reported; therefore, we attempted several centrifugation conditions in a preliminary study. When the suspension of Pinellia Tuber powder in water was centrifuged at milder conditions less than 4.5 × *g* for 10 min, the intermingled starch grains were hardly removed. At more intensive conditions than 72 × *g* for 10 min, the raphides and starch grains were both precipitated. Finally, we adopted the condition for the low-speed centrifugation as 18 × *g* for 10 min to separate the raphides in the supernatant and starch grains in the residue (Fig. 1a, b). However, a considerable amount of the starch grains was still observed among the raphides in the supernatant of the low-speed centrifugation, and no increase in the degrees of purification was observed by the repeated re-suspension and re-centrifugation. Therefore, we applied the PEX protocol, which was once reported to isolate raphides from taro leaves [9]. A fair amount of the raphides almost free from the contamination of starch grains was easily obtained by the PEX protocol (Fig. 1c). Then, the PEX suspension was dried under the stream of air at room temperature and re-suspended in the purified water (Fig. 1d) to perform the gustatory test. As shown in Table 1A, it was found that very intense acridity was retained by the PEX raphides. It was also found that when the PE was added to the aqueous suspension of the raphides obtained by low-speed centrifugation, the raphides still migrated to the PE layer (data not shown).

**Table 1:** Results of the gustatory test

		++	+	–
A	PEX raphides	3		
B	Boiled raphides		1	2
C	Pinellia Tuber incubated with raw ginger juice	1	2	
D	Pinellia Tuber incubated with boiled ginger juice		3	
E	Hangekoubokuto immersion: salad oil treatment (–)	3		
F	Hangekoubokuto immersion: salad oil treatment (+)			3

A gustatory test was performed as described in the methods. The numbers represent the number of subjects who report the following taste: + + , acrid irritation on the oral mucosa was sensed as clear and intense; + , less intense acridity was sensed; and –, no taste

### The characteristics of the raphides of Pinellia Tuber

**Fig. 2:**
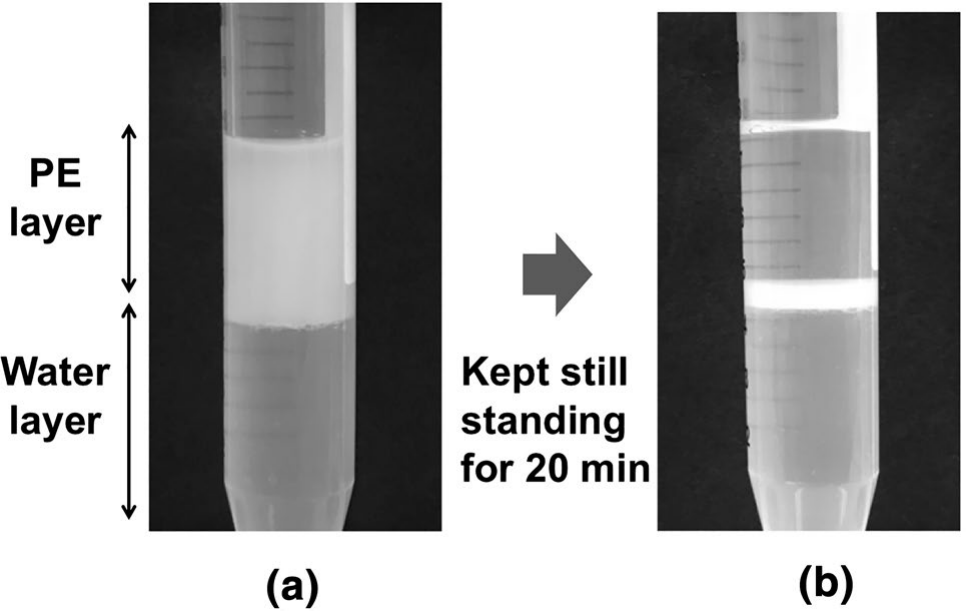
Lipophilicity of the raphides. Petroleum ether (3.6 ml), purified water (4 ml), and PEX suspension (0.4 ml) were combined in a tube, shaken vigorously (**a**), and stood for 20 min (**b**). The raphides were observed in the PE layer (**a**), and sank to the interfacial surface to generate a middle layer when the tube was stood for 20 min (**b**)

When PE and purified water were added together to the PEX suspension in a tube and shaken vigorously, almost all the raphides had dispersed in the PE layer (Fig. 2a), then kept standing for 20 min, the raphides in the PE layer slowly settled to the interfacial surface between water and PE layer to form the middle layer (Fig. 2b).

**Fig. 3:**
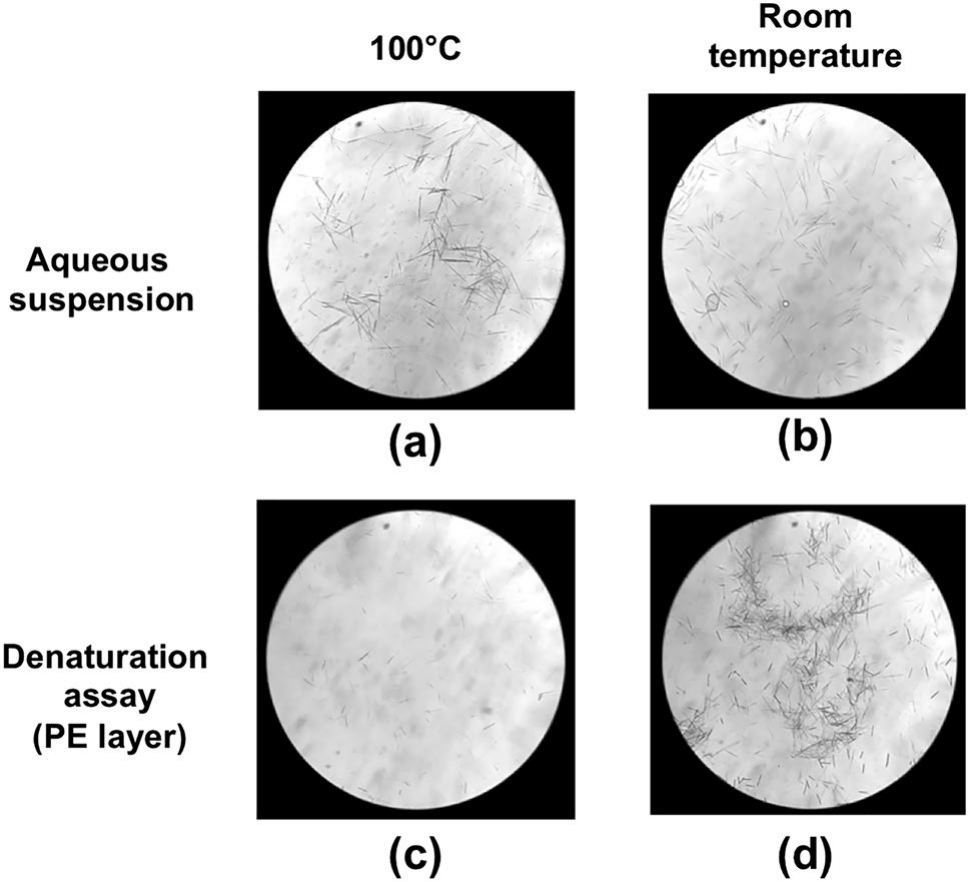
Denaturation of the raphides by heat. PEX suspension (0.4 ml) was dried and re-suspended in 5 ml of purified water, sealed in a tube, and incubated at 100 °C for 30 min (**a**) or at room temperature (**b**). The raphide pattern observed in the microscopic fields were very similar (**a** and **b**). Then, the denaturation assay described in “Materials and methods” was conducted. The results of the denaturation assay of (**a**) and (**b**) are shown in (**c**) and (**d**), respectively. Incubation at 100 °C for 30 min denatured the raphides, and the number of raphides dispersed in the PE layer was much lower in the sample incubated at 100 °C for 30 min (**c**) than in that without heating (**d**) in the microscopic field. The images were obtained using a light microscope at × 140 magnification

Subsequently, the PEX raphides were treated in various conditions to see their stability. First, the PEX suspension was dried by air-blowing at room temperature, and re-suspended in purified water. The aqueous raphide suspension was sealed in test tubes, and incubated for 30 min at 100 °C or at room temperature. The observation using light microscope showed no apparent differences between the boiled and unboiled raphides (Fig. 3a, b). However, when PE was added into these aqueous suspensions, the dispersion of the raphides in the PE layer of the boiled one was distinctly lower in the denaturation assay compared with that of the unboiled one (Fig. 3c, d). Furthermore, the acridity of the boiled raphides was almost imperceptible on the gustatory test (Table 1B).

**Fig. 4:**
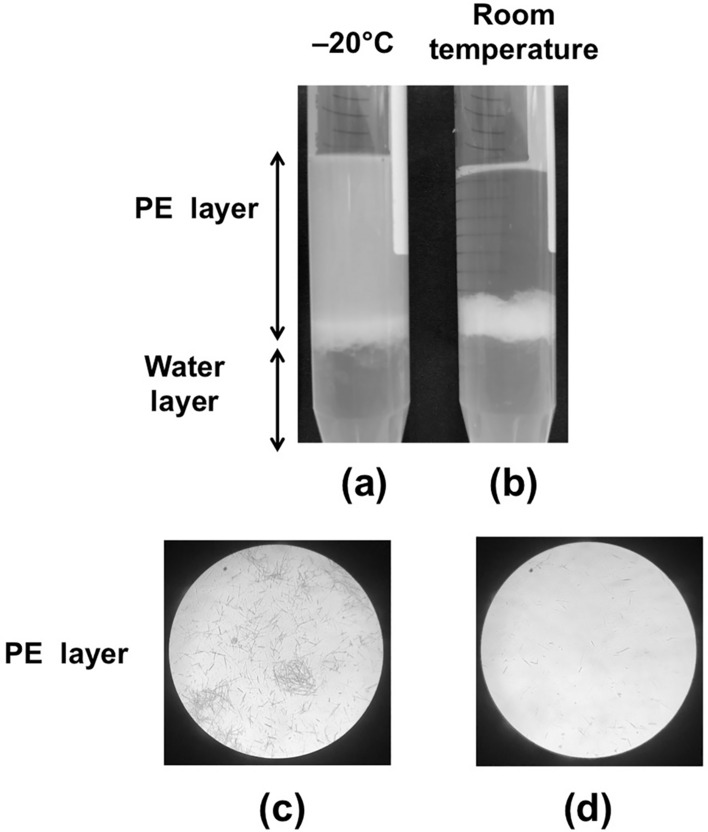
Denaturation of the raphides stored at room temperature. PEX suspension was kept for 60 days at − 20 °C (control) (**a**, **c**) or at room temperature (**b**, **d**). Then, the PEX suspension (0.4 ml) was sampled and mixed with PE (3 ml) and purified water (3.4 ml). The samples were then kept still standing for 15 min, then shaken lightly to raise the cloud of raphides (**a**, **b**). The dispersion of raphides in the PE layer was lower in the sample kept at room temperature than in the control. The PE layer (30 µl) was dropped onto a slide glass, dried, then observed using the light microscope at × 140 magnification (**c**, **d**). The number of raphides in the microscopic field was much lower in the sample kept at room temperature for 60 days than in the control

Some of the PEX suspension was kept in a sealed glass vial at room temperature or − 20 °C condition for 60 days. The dispersing raphides in the PE layer in the sample kept at room temperature were found to be lower in the denaturation assay compared with those stored at − 20 °C (Fig. 4).

**Fig. 5:**
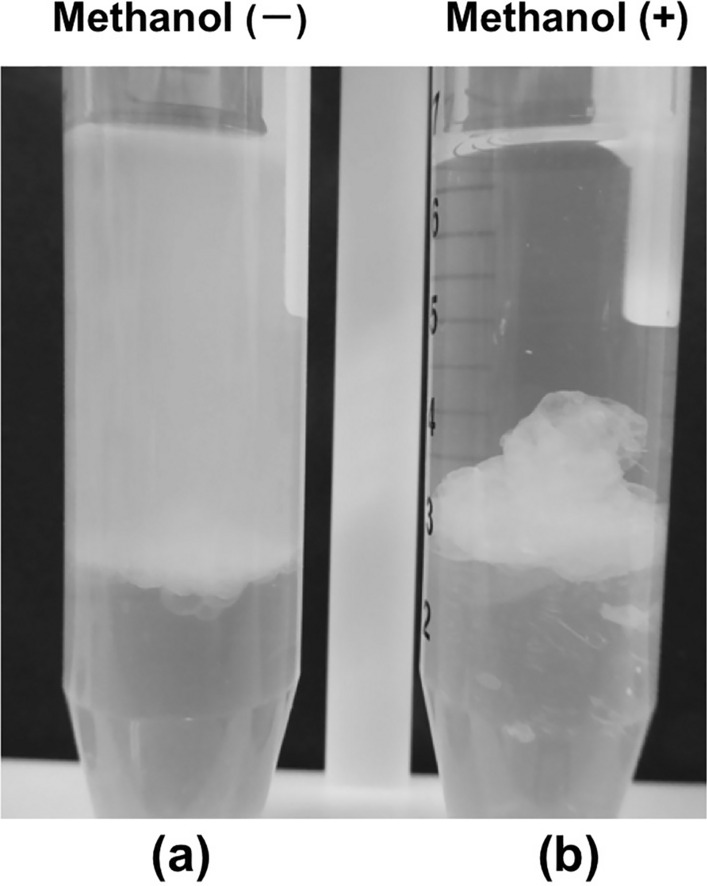
Denaturation of raphides by methanol. **a** PE (3 ml) and purified water (3 ml) were added to 0.4 ml of the PEX suspension, shaken, and stood for 10 s. **b** Methanol (6 ml) was added to 0.4 ml of the PEX suspension, mixed and centrifuged at 1.0 × 10^3^ × *g* to precipitate the raphides. The supernatant was removed and fresh methanol (6 ml) was added to the precipitate. After suspending in methanol, the sample was centrifuged again, and the supernatant was removed. Then, the precipitate was suspended in PE (6 ml) and centrifuged. After the precipitate was further washed with PE (3 ml) and the precipitate was suspended in fresh PE (3.4 ml), purified water (3 ml) was added to the suspension, and then the tube was shaken and stood for 10 s. Methanol treatment reduced the dispersion of raphides in PE layer, and the aggregated raphides quickly sank to the interfacial surface (**b**)

In another experiment, the PEX raphides were washed with methanol and re-suspended in PE. When water was added to this suspension and mixed vigorously, the raphides were aggregated and went down quickly to the interfacial surface without dispersing in PE layer (Fig. 5).

### Interactions between raphides and other crude drug extracts

**Fig. 6:**
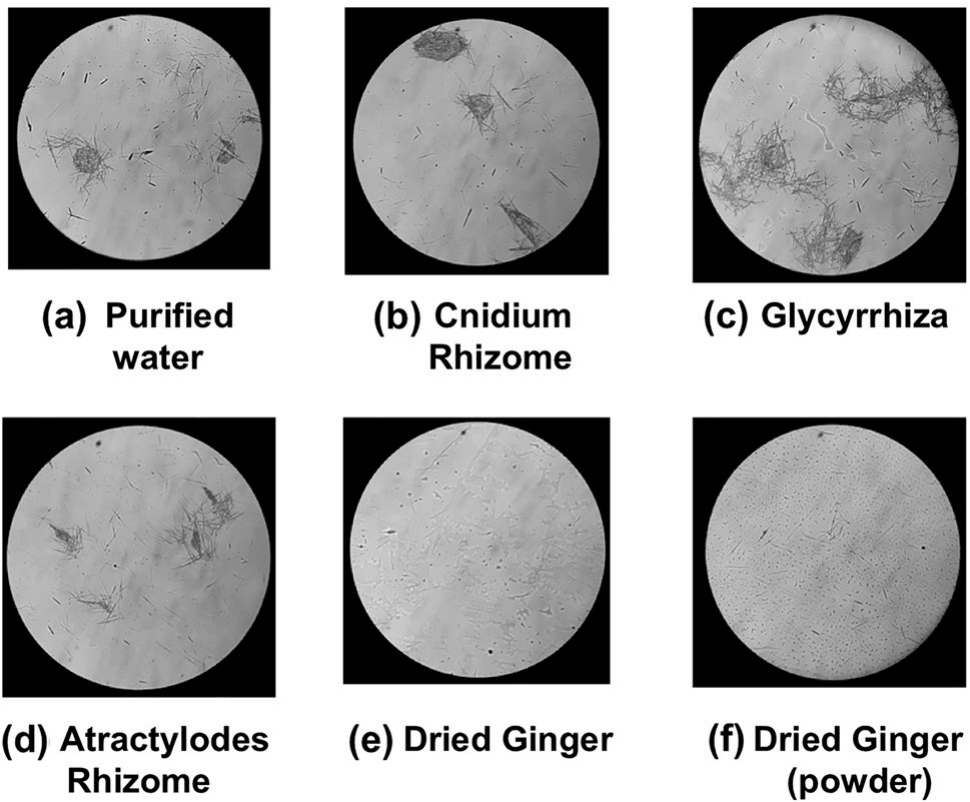
Interaction between raphides and other crude drug extracts. Two-milliliters each of purified water (control, **a**), decoctions of Cnidium Rhizome (**b**), Glycyrrhiza (**c**), Atractylodes Rhizome (**d**), dried Ginger (**e**), or 0.7 g of dried Ginger powder (**f**) were added to 0.7 g of Pinellia Tuber powder. Then, purified water was added to make the total volume to 6 ml, and incubated at 40 °C for 2 h. The raphides were collected according to materials and methods, and the denaturation assay described in “Materials and methods” was conducted. The raphides were observed in the microscopic field (**a**–**d**); the number of raphides was reduced by Ginger-treatment (**e** and **f**)

The decoction of Glycyrrhiza, Cnidium Rhizome, Atractylodes Rhizome, or dried Ginger was incubated with Pinellia Tuber powder at 40 °C for 2 h in the aqueous suspension. In another experiment, powdered Pinellia Tuber was incubated with powdered dried Ginger. Then, the raphides were collected from the reaction mixture. The denaturation assay showed that the decoction and the powder of dried Ginger reduced the dispersion of the raphides in the PE layer (Fig. 6e, f), while no such activity was observed in the decoctions of Glycyrrhiza, Cnidium Rhizome, and Atractylodes Rhizome (Fig. 6b–d).

**Fig. 7:**
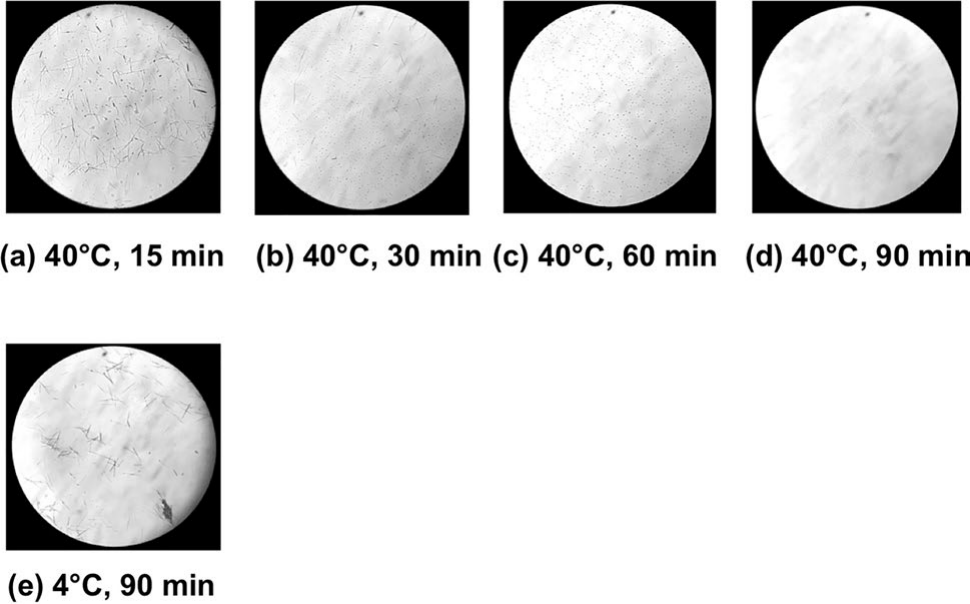
Reaction of the raphides with Ginger decoction. Dried Ginger decoction (2 ml) was added to Pinellia Tuber powder (0.7 g), and purified water was added to make the total volume up to 6 ml. The sample was incubated at 40 °C for 15 (**a**), 30 (**b**), 60 (**c**), and 90 min (**d**), respectively, or at 4 °C for 90 min (**e**). Then, a denaturation assay was conducted as described in “Materials and methods”. When the samples were incubated at 40 °C, the number of raphides in the microscopic field was reduced in a time-dependent manner (**a**–**d**). This reduction was not observed when the sample was incubated at 4 °C (**e**)

**Fig. 8:**
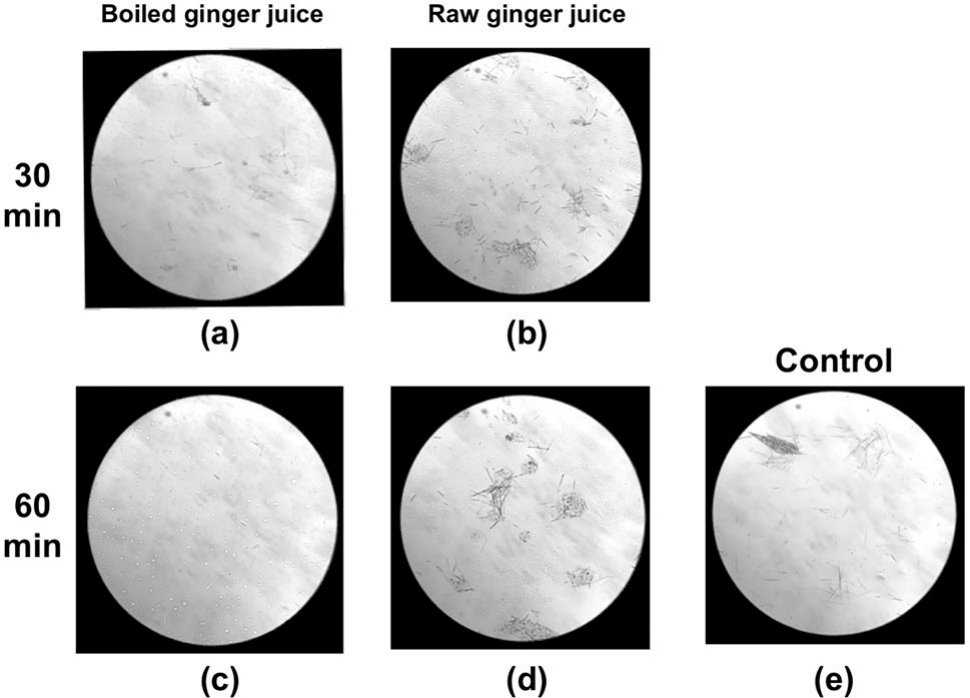
Difference in denaturing activity between raw and boiled ginger juice. Pinellia Tuber powder (0.7 g) was mixed with raw ginger juice (6 ml) (**a**, **c**), boiled ginger juice (6 ml) (**b**, **d**), or purified water (6 ml) (control, **e**), and incubated at 40 °C for 30 min (**a**, **b**) or 60 min (**b**, **d**, **e**). Then, a denaturation assay was conducted as described in “Materials and methods”. The number of raphides in the microscopic field was not changed following treatment with raw ginger juice (**b** and **d**) compared with the control (**e**); however, a reduction was observed in a time-dependent manner following treatment with boiled ginger juice (**a** and **c**)

**Fig. 9:**
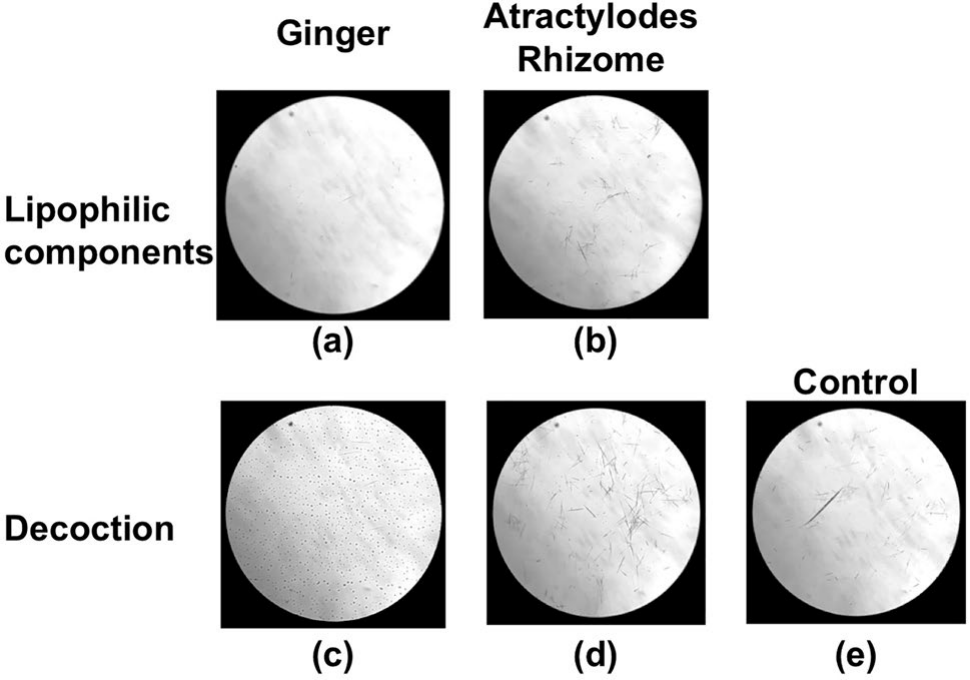
Interaction between Pinellia Tuber powder and the lipophilic components of dried Ginger and Atractylodes Rhizome. Pinellia Tuber powder (0.7 g) was transferred to a tube containing lipophilic components of Ginger (**a**) or Atractylodes Rhizome (**b**) prepared according as described in the Materials and Method, the decoctions (2 ml) of dried Ginger (**c**) or Atractylodes Rhizome (**d**), or purified water (2 ml) (control, **e**). Then, purified water was added to the tubes for a total volume of 6 ml. The mixtures were incubated at 40 °C for 2 h. Then, the denaturation assay described in “Materials and methods” was conducted. The number of raphides in the microscopic field was reduced by following treatment with lipophilic components of dried Ginger decoction (**a**) and its original decoction (**c**) compared with the control (**e**). No activity was observed in the lipophilic components from the Atractylodes Rhizome decoction (**b** and **d**)

Based on these results, the interaction between raphides and Ginger was investigated in more detail. When Pinellia Tuber powder and dried Ginger decoction were mixed and incubated at 40 °C for 15–60 min, time-dependent denaturation of the raphides was observed (Fig. 7a–d). Conversely, when incubation was performed at 4 °C, a considerable amount of the raphides dispersed in the PE layer after 90 min incubation (Fig. 7e). In another experiment, Pinellia Tuber powder was mixed with raw ginger juice or boiled ginger juice and incubated at 40 °C for 30–60 min. The boiled ginger juice denatured the raphides to a greater level than the raw juice (Fig. 8). A distinct decrease in acridity was also observed in the samples incubated with boiled ginger juice during the gustatory test (Table 1C and D). Subsequently, the lipophilic components extracted with PE from the decoction of dried Ginger or Atractylodes Rhizome were mixed with Pinellia Tuber powder in purified water and incubated at 40 °C for 2 h. Lipophilic components from the dried Ginger decoction, as well as its decoction, denatured the raphides; this activity was not observed with the lipophilic components from the Atractylodes Rhizome decoction (Fig. 9).

**Fig. 10:**
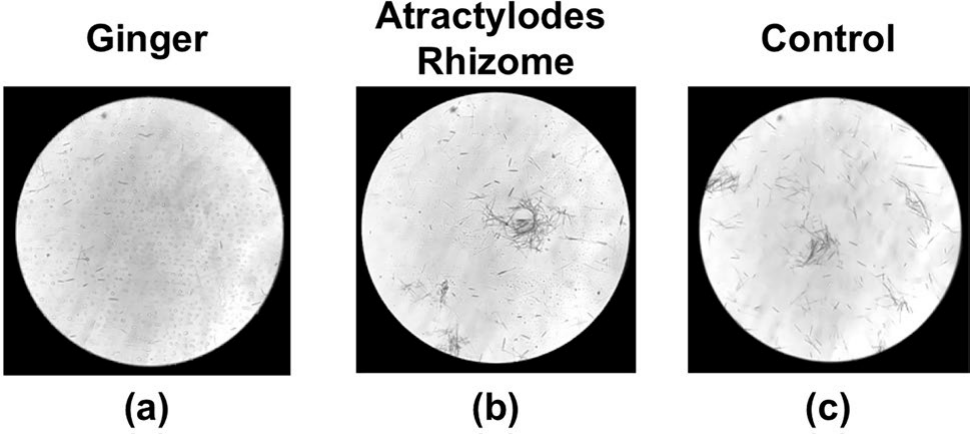
Interaction between the PEX raphides and the lipophilic components of Ginger and Atractylodes Rhizome. PEX suspension (0.4 ml) was dried and re-suspended in 4 ml of purified water then transferred to the tube containing the lipophilic components of Ginger (**a**) or Atractylodes Rhizome (**b**) prepared according as described in “Materials and methods”, or a control tube (**c**). The mixtures were incubated at 40 °C for 2 h. Then, a denaturation assay was conducted as described in “Materials and methods”. The number of raphides in the microscopic field was reduced following treatment with lipophilic Ginger components (**a**) compared with the control (**c**); however, this was not observed with the lipophilic components of the Atractylodes Rhizome decoction (**b**)

Finally, PEX suspension was dried by air-blowing at room temperature, and re-suspended in purified water. This suspension was mixed with the lipophilic components from dried Ginger or Atractylodes Rhizome decoction, and incubated at 40 °C for 2 h. Again, the lipophilic components from dried Ginger decoction possessed denaturing activity against the purified raphides as observed in the experiment using Pinellia Tuber powder (Fig. 10). In addition, even a very small amount of PE in the test tube impeded the denaturing activity of the dried Ginger decoction (data not shown).

### Alleviation of the irritation by salad oil

**Fig. 11:**
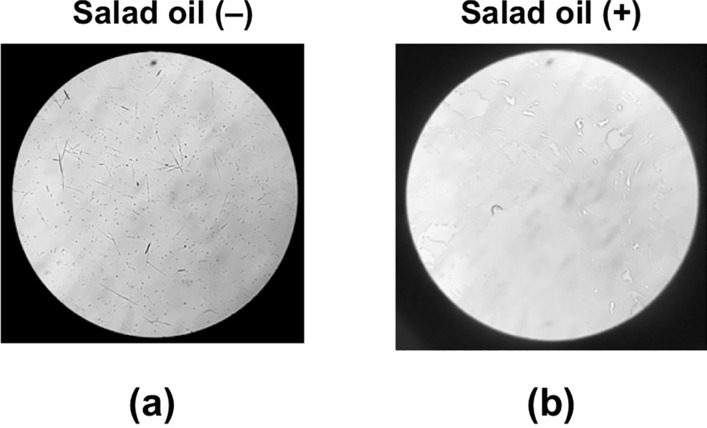
Treatment of Hangekoubokuto IPCD immersion with salad oil. Hangekoubokuto immersion was prepared by the IPCD method as described in “Materials and methods” (**a**). Salad oil (5 ml) was added to the immersion (5 ml) and mixed vigorously. Then, the water layer was collected (**b**). Images were obtained using a light microscope at × 140 magnification. The number of raphides in the microscopic field was reduced following treatment with salad oil (**b**) compared with the control (**a**)

Hangekoubokuto immersion was prepared with the IPCD method using unprocessed Pinellia Tuber. The immersion was mixed vigorously with an equal volume of salad oil, then the water layer was collected. Very few raphides were observed in the water layer of the Hangekobokuto immersion (Fig. 11) and a distinct decrease in acridity was observed following treatment with salad oil (Table 1E and F). In addition, in volunteers, acrid irritation of the oral mucosa induced by Pinellia Tuber was relieved by rinsing the mouth with the 5 ml of salad oil.

## Discussion

Adopting the PEX protocol, raphides were easily extracted from Pinellia Tuber. Hydrophilic components, such as starch grains, remained in the aqueous residue, and most of the lipophilic components which were soluble in PE, were washed away during the extraction process. In fact, contamination of starch grains was very limited among the raphides in the PEX suspension when observed under a light microscope; thus, the PEX protocol is suggested to be a valid method for the partial purification of raphides in Pinellia Tuber. It is also noted that while PEX raphides retained intense acridity, homogentisic acid and 3,4-dihydroxybenzaldehyde were likely to be solved and removed in PEX protocol, which was consistent with the previous works reporting the raphides were the cause of the acridity [7, 8]. Since the raphides extracted by low-speed centrifugation in aqueous suspension also partitioned to the PE layer in the water/PE partition, most of raphides in Pinellia Tuber were considered to have lipophilic character. When calcium oxalate was mixed vigorously with water and PE, it migrated only to the water layer (data not shown). Therefore, the substance associated with the lipophilicity of the raphides was not calcium oxalate. It has not been cleared yet how the raphides migrated to the PE layer whereas they had originally been extracted using water. Our recent results showed the possibility that the acrid raphides suspended in water could switch their hydrophilic character to lipophilic when they touched nonpolar substances. The confirmation of this hypothesis is now in progress, and the authors will report it elsewhere.

In this study, the dispersion of denatured raphides was reduced in the PE layer in the water/PE partition. No apparent differences were observed between the behavior of the intact and the denatured raphides when they were suspended in either PE or water alone. Differences appeared only in the presence of both PE and water, suggesting that denaturation can decrease their lipophilicity to a certain degree.

Based on this result, we developed a novel protocol for the denaturation assay of raphides, as described in the methods section. In the present study, raphides were shown to be denatured by heat, methanol, dried Ginger extract, and boiled ginger juice. As no apparent differences were observed between boiled and non-boiled raphides under the light microscope, it was thought that the substances denatured by heat were not the components responsible for formation of the raphides’ shape but something attached on the surface of the crystals. This was consistent with a previous observation of deposits on the surface of taro raphides, where the amount of deposits, as well as the acridity, decreased following treatment with methanol [9]. In this study, we also found that the raphides from Pinellia Tuber were markedly denatured by the methanol wash, and speculate that the lipophilic deposits could be removed by methanol as reported in taro. The raphides kept in PE at room temperature were denatured to a larger degree compared with those stored at − 20 °C, suggesting that raphides in the dried Pinellia Tuber can also be gradually denatured under natural storage conditions. In the sixth century, Tao Hong Jing selected six crude drugs, including Pinellia Tuber; of these, the older drugs were conforming items compared with new ones in *Bencaojingjizhu* [12]. This could be related to the denaturation of raphides stored at room temperature, as observed in the present study, from the point of reducing adverse events, however, the confirmation of this hypothesis still requires the comparison of the raphides in fresh and aged dried Pinellia Tuber, which is now in progress.

The acridity assay is crucial for investigating the safe and effective use of Pinellia Tuber. The gustatory test provides distinct results on acridity, but it forces the subjects to experience some degree of pain. The acridity of the taro raphides was previously assayed by applying samples to the forearm of volunteers [9]. The same protocol was attempted here, but no distinct or reproducible results were obtained, possibly because of differences in the structures of the raphides between taro and Pinellia Tuber. The acridity of Pinellia Tuber was also assayed by applying samples to the eyes of rabbits to assess inflammation [7]. However, this protocol involves animal experimentation, which is difficult to perform under current scientific ethics. In this study, raphide dispersibility in the PE layer in the water/PE partition paralleled the results of the gustatory tests, suggesting the development of a novel and convenient assay protocol.

Tao Hong Jing also noted that Pinellia Tuber must be used with Ginger to suppress its poisonous nature [13]; thus, the suppression of Pinellia Tuber acridity by Ginger has been widely recognized, but has not been well studied. In this study, Ginger was shown to have specific activity in the denaturation of Pinellia tuber raphides. This activity was shown to require a certain reaction temperature and a time of more than 30 min. This observation is consistent with the fact that Pinellia Tuber detoxification has been conducted effectively for many years in China by immersing in ginger juice or alum. Conversely, drinking ginger juice is ineffective at relieving the pain induced by unprocessed Pinellia Tuber; this is because the juice may have insufficient time to denature the raphides in the mouth and throat.

The requirements for a certain reaction temperature and reaction time are consistent with enzymatic reactions. However, interestingly, the activity of Ginger was reinforced by boiling the juice. Therefore, it is unlikely that the main activity was induced by a protein. The observation that the PEX raphides were denatured by the extracted lipophilic components of Ginger alone may suggest that denaturation resulted from the direct interaction between raphides and the lipophilic components of Ginger. For example, shogaol is a lipophilic component of Ginger, whose content increases by heat [14]. However, we have not identified the reacting component of Ginger nor clarified the mechanism by which it denatures the raphides. These problems await further research.

The IPCD method was developed for easier preparation of Kampo decoctions. In the IPCD method, the crude drugs are powdered and mixed vigorously with boiled water then filtered after 4 min [11]. This method is convenient; however, the short heating time results in intense residual acridity of the unprocessed Pinellia Tuber, which is a major problem. In this study, we successfully removed the acridity of IPCD immersion using unprocessed Pinellia Tuber by mixing with salad oil, and collecting the water layer. It is supposed that the acrid raphides in water layer can transfer into the interfacial surface between water and salad oil layer as observed in water/PE partition, and can be removed by collecting water layer. Mixing salad oil may indicate how unprocessed Pinellia Tuber can be used safely in the IPCD method.

In addition, rinsing the mouth with salad oil is an effective way of relieving the irritation. This will, for example, greatly reduce the pain experienced by volunteers during the gustative bioassay when it has to be carried out.

In conclusion, **t**he raphides of Pinellia tuber have lipophilic character and easily extracted using petroleum ether. Lipophilicity was thought to be derived from a substance attached to the surface of the raphides, which was denatured by heat, methanol, and Ginger extract. The denaturation of raphides by Ginger extract may explain the traditional protocol used to process Pinellia tuber. Furthermore, denaturation of the raphides seemed to parallel their reduced acridity, which could lead to the development of a new assay protocol to assess acridity.

## Electronic supplementary material

Below is the link to the electronic supplementary material.

Supplementary file 1 (PDF 1162 kb)

## References

[1] Pharmaceutical and Medical Device Regulatory Science Society of Japan (2017). Japanese pharmacopoeia seventeenth edition (JP XVII) english version.

[2] The Japanese Society for Oriental Medicine (2005). Introduction to kampo, Japanese traditional medicine.

[3] Chinese Pharmacopoeia Commission (2015). The 2015 edition of pharmacopoeia of the People’s Republic of China.

[4] Bensky D, Clavey S, Stöger E (2004). Chinese herbal medicine: materia medica.

[5] Cai B (2008). Zhongyaopaozhixue.

[6] Wu H, Li W, Han H, Ji R, Ye D (1999). Studies on stimulating components of raw *Pinellia ternata* (Thunb.)(Banxia). Chin J Chin Mater Med.

[7] Zhong L, Wu H, Zhang K, Wang Q (2006). Study on irritation of calcium oxalate crystal in raw *Pinellia ternata*. Chin J Chin Mater Med.

[8] Wu H, Zhong L, Zhang L, Zhu T (2007) The study on the toxicity of the calcium oxalate raphide in Pinellia Tuber and its binding protein. Proceedings of the 5th Academic Conference of 2nd session and the 3rd Member Congress for Four Famous Crude drugs of Huaiqing Research Forum in China Association of Chinese Medicine, Processing of Chinese Medicine Branch

[9] Paull ER, Tang C, Gross K, Uruu G (1999). The nature of the taro acridity factor. Postharvest Biol Technol.

[10] Wu H, Yu H, Ge X, Pan H, Jing Y, Zhan Q (2014) The common detoxification processing mechanism of poisonous *Araceae* herbal medicines. China Ninxia Ethnic Medicine International Forum, pp. 301–312

[11] Fueki T, Makino T, Matsuoka T, Beppu M, Sunaga T, Tanaka K, Nagamine K, Namiki T (2015). Quick and easy preparation method for decoction of Kampo formula inspired by the method of boiling powdered crude drugs in the Song period of China. Tradit Kampo Med.

[12] Tao H (1997) The first *Xulu* of *Bencaojizhu.* In Research Institute for Buddist Culture (ed) *Bencaojizhuxulu Biqiuhanzhujie*, Dunhuang's Manuscript, Facsimile Series of Rare Texts in the Library of Ryukoku University, No. 16. Hozokan, Kyoto, p 54

[13] Tao H, Tan S, Shang Z, Zheng J, Shang Y, Liu D (1993). Banxia. Zhengleibencao—Zhongxiuzhenghejingshizhengleibeiyongbencao.

[14] Yoshida M, Hirabayashi S (2015). Changes in 6-gingerol concentration in ginger under various types of cooking conditions. J Cook Sci Jpn.

