# Correction to: The acrid raphides in tuberous root of *Pinellia ternata* have lipophilic character and are specifically denatured by ginger extract

**DOI:** 10.1007/s11418-021-01501-5

**Published:** 2021-03-06

**Authors:** Tsukasa Fueki, Koichiro Tanaka, Kunihiko Obara, Ryudo Kawahara, Takao Namiki, Toshiaki Makino

**Affiliations:** 1 Matsuya Pharmacy, Niigata, Japan; 2 Department of Traditional Medicine, Toho University School of Medicine, Tokyo, Japan; 3 Department of Pharmacognosy, Graduate School of Pharmaceutical Sciences, Nagoya City University, Nagoya, Japan; 4 Obara Coloproctology Clinic, Tokyo, Japan; 5 Department of Cardiology, Tokyo Nishi Tokushukai Hospital, Tokyo, Japan; 6 Department of Japanese-Oriental (Kampo) Medicine, Graduate School of Medicine, Chiba University, Chiba, Japan

## Correction to: Journal of Natural Medicines (2020) 74:722–731 10.1007/s11418-020-01425-6

The article The acrid raphides in tuberous root of *Pinellia ternata* have lipophilic character and are specifically denatured by ginger extract, written by Tsukasa Fueki, Koichiro Tanaka, Kunihiko Obara, Ryudo Kawahara, Takao Namiki and Toshiaki Makino, was originally published Online First without Open Access. After publication in volume 74, issue 4, page 722–731 the author decided to opt for Open Choice and to make the article an Open Access publication. Therefore, the copyright of the article has been changed to © The Author(s) 2021 and the article is forthwith distributed under the terms of the Creative Commons Attribution 4.0 International License (https://creativecommons.org/licenses/by/4.0/), which permits use, sharing, adaptation, distribution and reproduction in any medium or format, as long as you give appropriate credit to the original author(s) and the source, provide a link to the Creative Commons license, and indicate if changes were made.

The original article has been updated.

