# Supplementary material for: The acrid raphides in tuberous root of Pinellia ternata have lipophilic character and are specifically denatured by ginger extract
Source: J Nat Med. 2020 Jun 26;74(4):722–31. doi: 10.1007/s11418-020-01425-6 (PMC7929962; doi:10.1007/s11418-020-01425-6)
Supplement: Supplementary file 1 — (PDF 1162 kb) [file 11418_2020_1425_MOESM1_ESM.pdf]

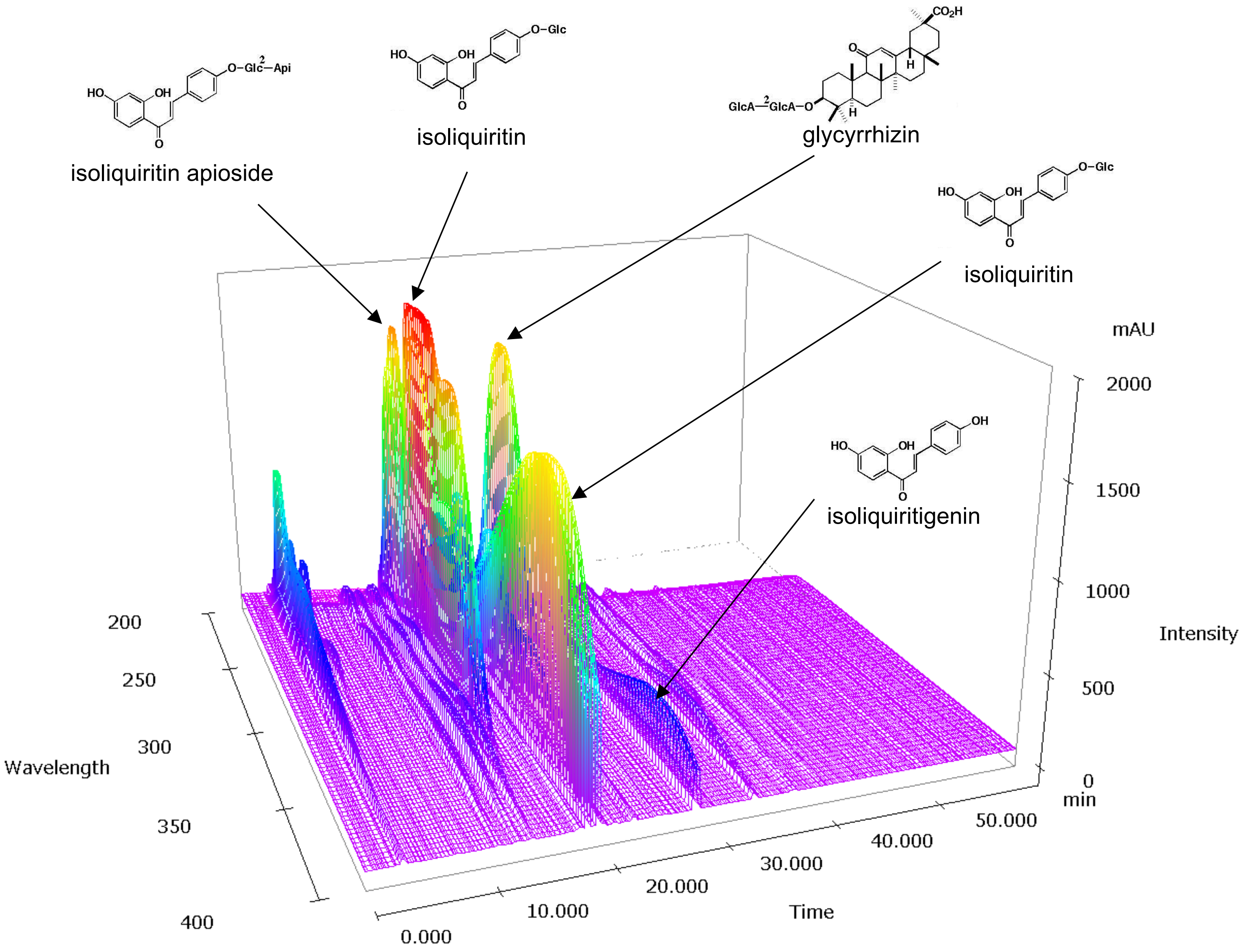

Supplemental Fig. 1
The extract of kanzo (25 mg) was suspended with MeOH (1 ml) and sonicated for 30 min. The supernatant (25 µl) was injected to HPLC with the following conditions: system, Shimadzu LC–10A$_{VP}$ (Kyoto, Japan); column, TSK–GEL ODS–80$_{TS}$ (4.6 × 250 mm, Tosoh, Tokyo); mobile phase, 0.05 M AcOH–AcONH$_4$ buffer (pH 3.6)/CH$_3$CN 90:10 (0 min) – 0:100 (60 min), linear gradient; flow rate, 1.0 ml/min; column temperature, 40°C; and detection, 200 – 400 nm by a photodiode array detector. Some peaks were identified by the retention times and UV spectra of the standard compounds.

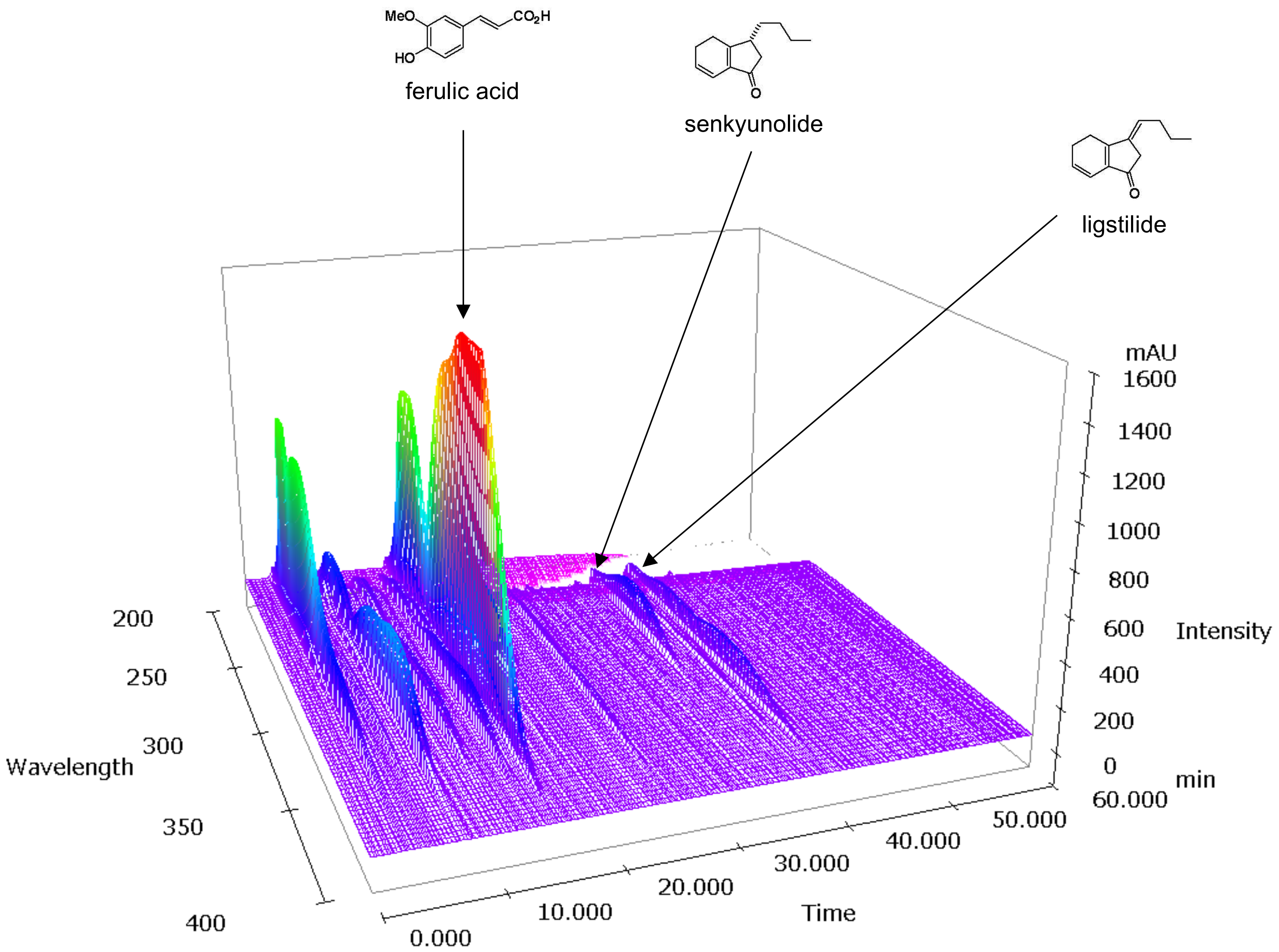

Supplemental Fig. 2
The extract of senkyu (25 mg) was suspended with MeOH (1 ml) and sonicated for 30 min. The supernatant (25 µl) was injected to HPLC with the following conditions: system, Shimadzu LC–10A$_{VP}$ (Kyoto, Japan); column, TSK–GEL ODS–80$_{TS}$ (4.6 × 250 mm, Tosoh, Tokyo); mobile phase, 0.05 M AcOH–$AcONH_4$ buffer (pH 3.6)/CH_3CN 90:10 (0 min) – 0:100 (60 min), linear gradient; flow rate, 1.0 ml/min; column temperature, 40°C; and detection, 200 – 400 nm by a photodiode array detector. Some peaks were identified by the retention times and UV spectra of the standard compounds.

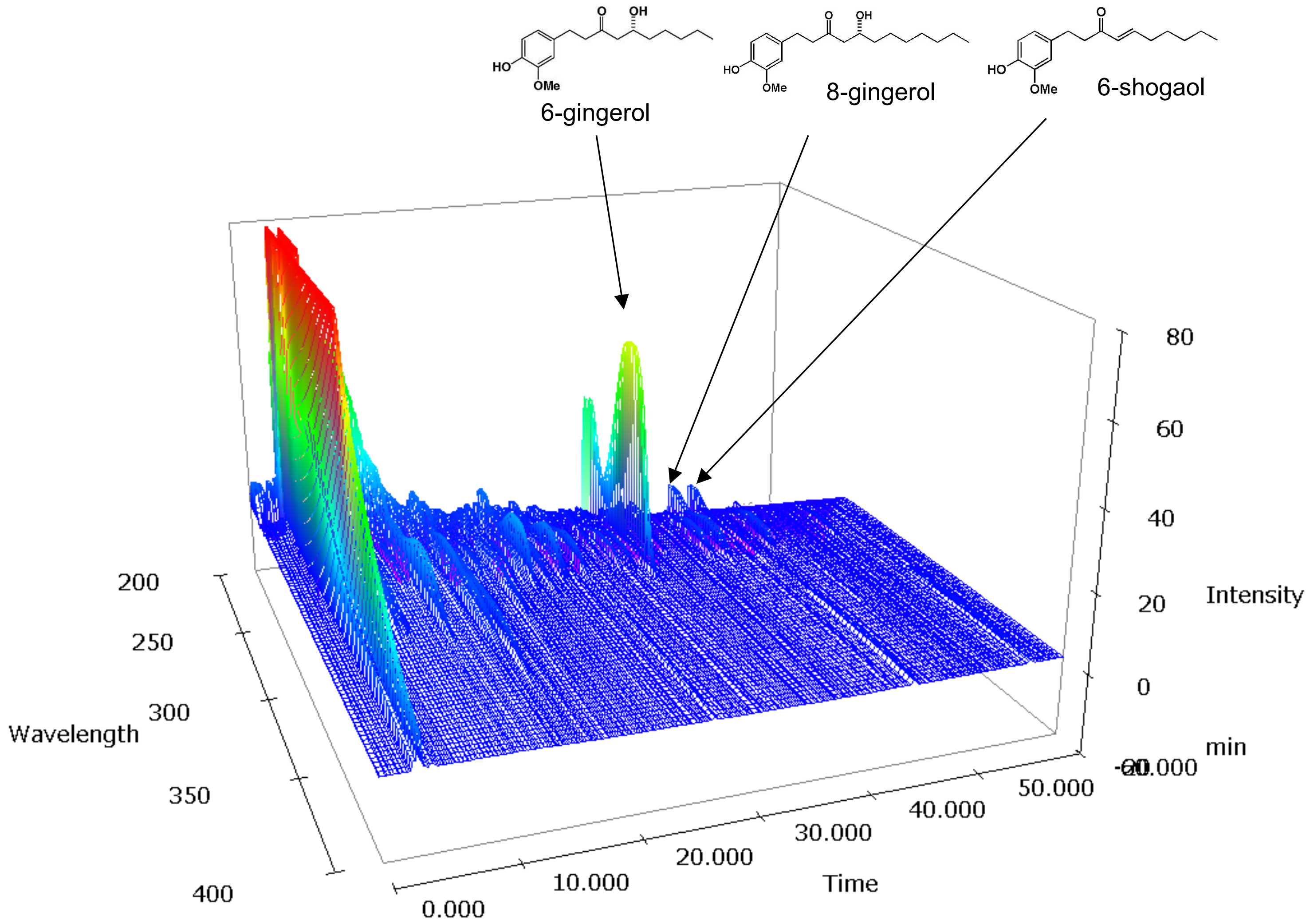

Supplemental Fig. 3
The extract of shokyo was suspended with MeOH (1 ml) and sonicated for 30 min. The supernatant (25 µl) was injected to HPLC with the following conditions: system, Shimadzu LC–10A$_{VP}$ (Kyoto, Japan); column, TSK–GEL ODS–80$_{TS}$ (4.6 × 250 mm, Tosoh, Tokyo); mobile phase, 0.05 M AcOH–AcONH$_4$ buffer (pH 3.6)/CH$_3$CN 90:10 (0 min) – 0:100 (60 min), linear gradient; flow rate, 1.0 ml/min; column temperature, 40°C; and detection, 200 – 400 nm by a photodiode array detector. Some peaks were identified by the retention times and UV spectra of the standard compounds.

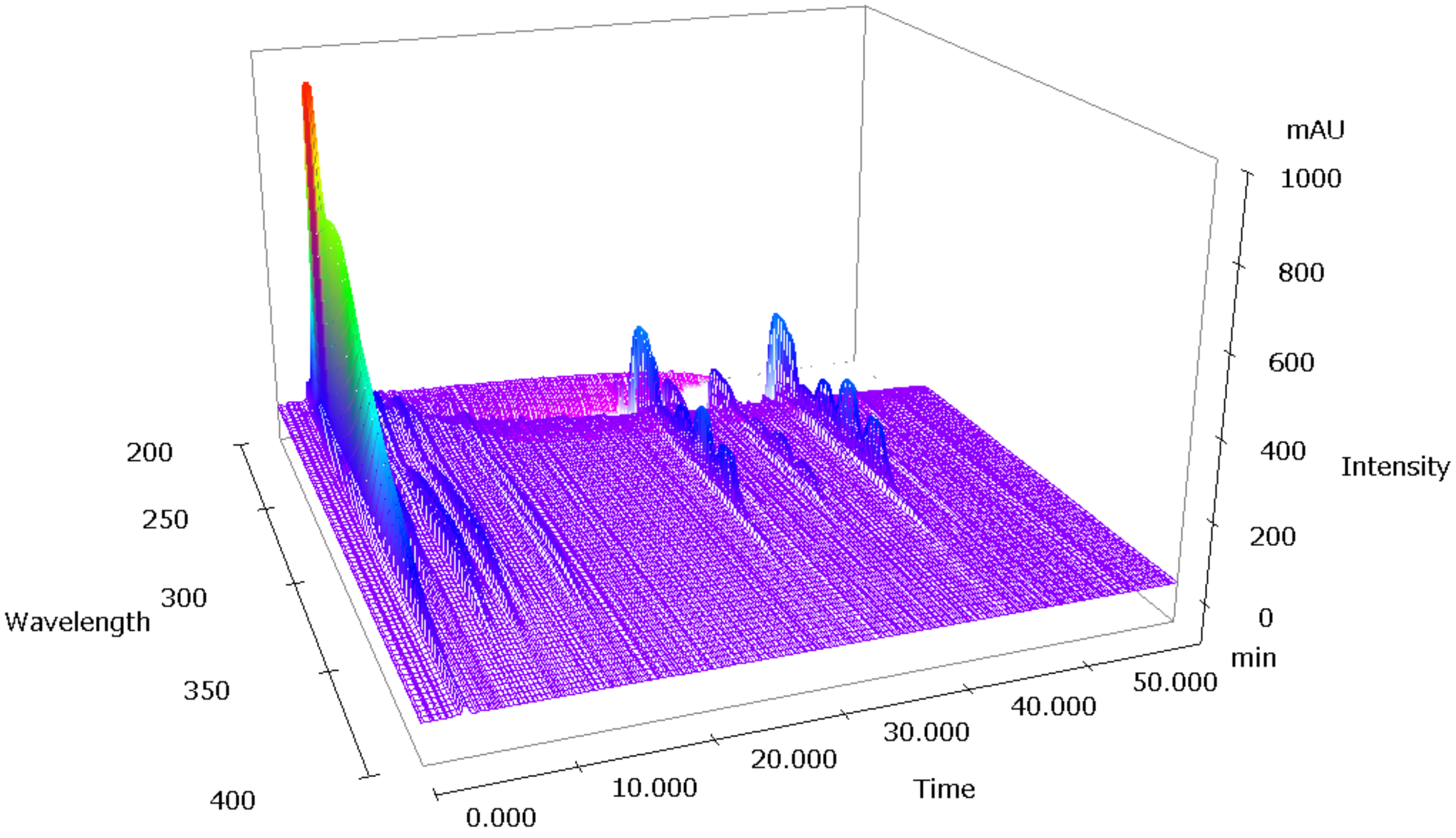

Supplemental Fig. 4
The extract of byakujutsu (25 mg) was suspended with MeOH (1 ml) and sonicated for 30 min. The supernatant (25 µl) was injected to HPLC with the following conditions: system, Shimadzu LC–10A$_{VP}$ (Kyoto, Japan); column, TSK–GEL ODS–80$_{TS}$ (4.6 × 250 mm, Tosoh, Tokyo); mobile phase, 0.05 M AcOH–$AcONH_4$ buffer (pH 3.6)/CH_3CN 90:10 (0 min) – 0:100 (60 min), linear gradient; flow rate, 1.0 ml/min; column temperature, 40°C; and detection, 200 – 400 nm by a photodiode array detector. Some peaks were identified by the retention times and UV spectra of the standard compounds.

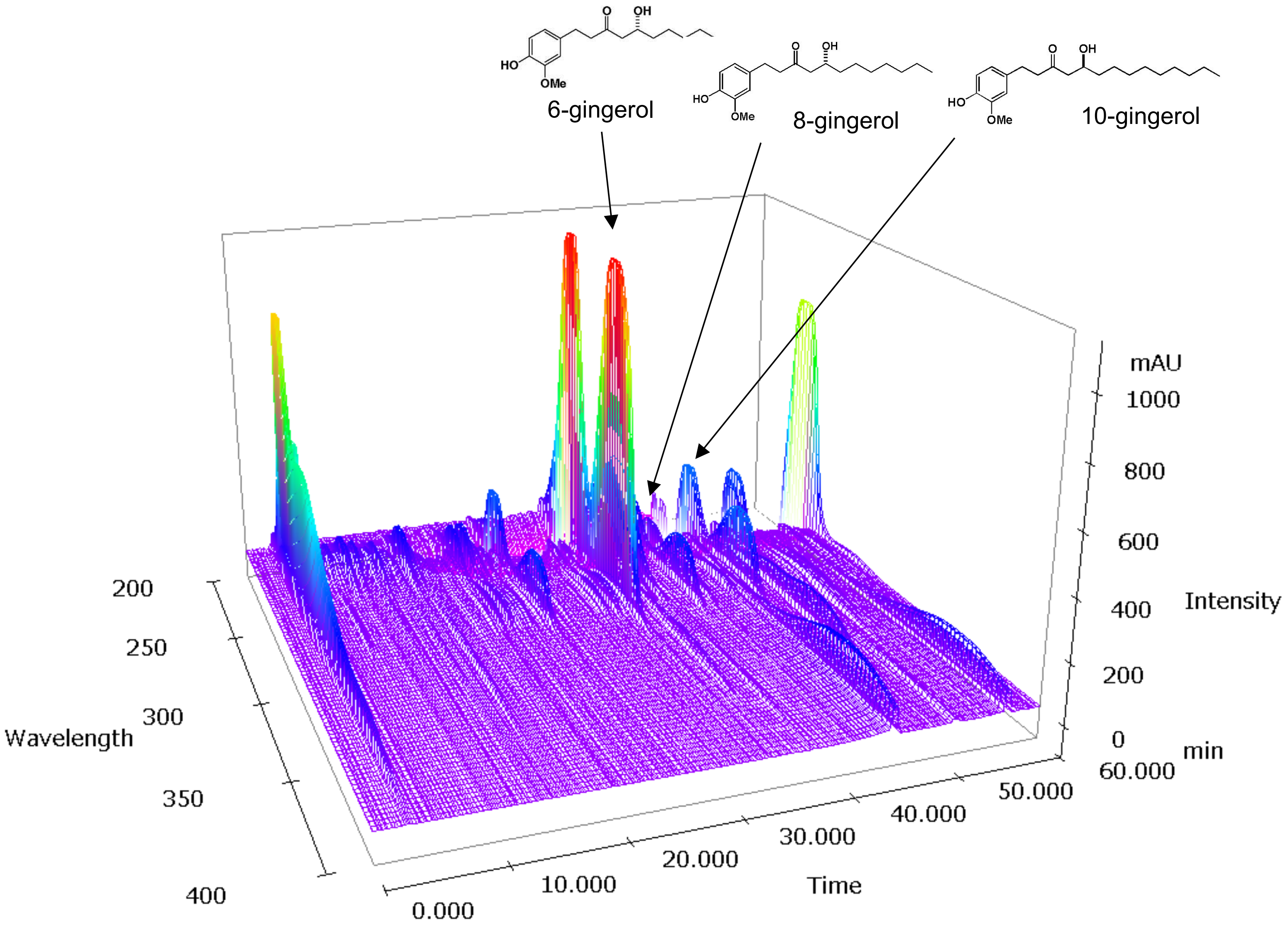

Supplemental Fig. 5
The extract of fresh shokyo (25 mg) was suspended with MeOH (1 ml) and sonicated for 30 min. The supernatant (25 μl) was injected to HPLC with the following conditions: system, Shimadzu LC–10A$_{VP}$ (Kyoto, Japan); column, TSK–GEL ODS–80$_{TS}$ (4.6 × 250 mm, Tosoh, Tokyo); mobile phase, 0.05 M AcOH–AcONH$_4$ buffer (pH 3.6)/CH_3CN 90:10 (0 min) – 0:100 (60 min), linear gradient; flow rate, 1.0 ml/min; column temperature, 40°C; and detection, 200 – 400 nm by a photodiode array detector. Some peaks were identified by the retention times and UV spectra of the standard compounds.
